# Determinants of Cervical screening services uptake among 18–49 year old women seeking services at the Jaramogi Oginga Odinga Teaching and Referral Hospital, Kisumu, Kenya

**DOI:** 10.1186/1472-6963-14-335

**Published:** 2014-08-06

**Authors:** Everlyne N Morema, Harrysone E Atieli, Rosebella O Onyango, Joyce H Omondi, Collins Ouma

**Affiliations:** Department of Public Health, Maseno University, Private Bag, Maseno, Kenya; Kenya Medical Research Institute/Centre for Global Health Research, Kisumu, Kenya; Department of Biomedical Sciences and Technology, Maseno University, Private Bag, Maseno, Kenya

**Keywords:** Cervical, Women, Screening services, Determinants, Kenya

## Abstract

**Background:**

Kenyan women aged ≥15 years are at risk of developing cervical cancer. Currently, cervical cytology reduces cervical cancer incidence, since it allows for early diagnosis and treatment. Uptake of cervical screening services is a priority research area in Kenya. Central to the success of any screening programme is its ability to identify, reach out and screen the defined target population. Cervical screening coverage in Kenya is currently at 3.2%. In Jaramogi Oginga Odinga Teaching and Referral Hospital (JOOTRH) in Nyanza, the number screened for cervical cancer is low (averagely 3/day). Thus the current study sought to identify factors influencing uptake of cervical screening services at the facility.

**Methods:**

In a cross-sectional study, knowledge, perceptions and cues for action associated with self-reported cervical screening uptake were explored. The targeted population (n = 424), purposively selected were women of child-bearing age (18–49 years) visiting JOOTRH. Data on socio-demographic status (age, level of education, marital status, job status, income level), knowledge of cervical cancer, perceptions on severity and susceptibility to the disease were collected using self-administered structured questionnaires. Statistical significance of differences in proportions were determined by chi-square analyses while logistic regression analyses were used to identify determinants of self-reported uptake of the service.

**Results:**

Self-reported screening uptake was 17.5%. There was a strong positive association between age (*P* < 0.0001), level of education (*P* < 0.0001) and income levels (*P* = 0.005) with the uptake of the service. Knowledge level on the signs and symptoms of cervical cancer was an important determinant for being screened for cervical cancer (*P* < 0.0001). Furthermore, those who said they didn’t know about the disease (OR, 26.84, 95% CI, 6.07-118.61, *P* < 0.0001) or were not aware about susceptibility to it (OR, 2.37, 95% CI, 1.10-5.08, *P =* 0.02) had a higher likelihood of not being screened. On cues for action, those who attended the child welfare clinic were more likely to be screened (OR, 2.31, 95% CI, 1.17-3.93, *P =* 0.03).

**Conclusion:**

Knowledge, perception of higher susceptibility and attending child welfare clinic are key determinants of self-reported uptake of cervical screening. Increasing knowledge, enhancing health education and providing free services may increase uptake among women population in such settings.

**Electronic supplementary material:**

The online version of this article (doi:10.1186/1472-6963-14-335) contains supplementary material, which is available to authorized users.

## Background

Kenya has a population of 10.32 million women aged 15 years and older who are at risk of developing cervical cancer. Current estimates indicate that every year, 2454 women are diagnosed with cervical cancer with 1676 deaths resulting from it. Cervical cancer ranks as the second most frequent cancer among women in Kenya aged between 15 and 44 years of age. Documenting the scope of the problem is beset by the paucity of cancer registries in many low-resource countries, Kenya included [1].

Cervical cancer is a malignant neoplasm of the cervix uteri or cervical area. It may present with vaginal bleeding, but symptoms may be absent until the cancer is in its advanced stages. Treatment consists of surgery (including local excision) in early stages and chemotherapy and radiotherapy in advanced stages of the disease. If pre-malignant disease or cervical cancer is detected early through screening, it can be monitored or treated relatively non-invasively [2]. Central to the success of any screening programme is its ability to identify, reach and screen the defined target population [3].

A National Cervical Cancer Prevention Program with an action plan to run for 5 years was initiated by the Division of Reproductive Health, Ministry of Health Kenya in July 2005 [4]. The program aimed at 20% screening coverage in the 5 years the program was to run i.e. between 2005–2009. Unfortunately, this was not achieved at the end of the period. Thus uptake of cervical screening services was identified as a priority research area by the Division of Reproductive Health of Kenya in its research agenda for the period 2010–2014 [5]. Among the principles that guide Kenya as it implements its National Reproductive Health Strategy is the adoption of evidence-based reproductive health practices. Early detection and treatment is the only way to prevent deaths due to cervical cancer. Age standardized death rates for countries with high cervical screening like USA and Canada is 2.5 per 1,000 women, while in Kenya it is 28.7 (the total number of deaths per year per 1,000 people of a given age) [5]. In the developing world, women generally do not know they have this cancer until it is at a symptomatic and untreatable stage [1]. Any intervention to increase uptake of cervical screening must be tailored to the baseline knowledge, perceptions, culture, and attitudes unique to the target population [6]. Cervical screening coverage in Kenya is currently at 3.2% for all women, 4.0% and 2.6% for urban and rural women, respectively [5, 7]. This is way below the 25% and 75% projected by National Cervical Cancer Prevention Program, Action Plan 2005–2009, for the national coverage and coverage per district, respectively [4]. The focus of the cervical cancer program in Kenya is women aged 25–49 years. However, women outside this age group who request or for whom screening is recommended are not to be denied services [5]. The recommended screening cycle for the Kenya program is every 5 years, except for HIV positive women. All HIV positive women with history of sexual activity, and are 18–65 years old are to be screened for cervical cancer as part of comprehensive HIV care. The screening cycle for this category is at diagnosis, after every 6 months in year one and then yearly if normal [5]. A previous study on Knowledge, Attitude and Practices (KAP) carried out in Kenyatta National Hospital revealed a past Pap smear screening rate of 22% [8], while in a different study performed in Voluntary Testing and Counseling (VCT) centers in Nairobi, Kenya [9], an uptake rate of 14% was described. These demonstrate a relatively low level of uptake of cervical screening.

Studies carried out elsewhere show that various factors are associated with uptake of cervical cancer screening. For instance, in Britain, socio-demographic and attitudinal correlates of self-reported cervical screening uptake were investigated on a national sample in 1999 [10]. Of the socio-economic indicators, only age of completed full-time education showed a significant effect in the multivariate analysis [10]. Uptake was highest among married and separated women and lowest among single and widowed women [10]. Anticipated embarrassment and attitudes to screening (e.g. ‘There’s no point going for screening if you don’t have any symptoms”) were significant independent predictors of uptake [10]. A desk review aimed at highlighting the main predictors of participation in cervical screening programmes and interventions that can be used to increase cervical screening uptake revealed that age and ethnicity were factors affecting cervical screening participations [11]. Women in the highest occupational class had a higher likelihood of cervical screening compared to those in the lowest class [12]. At the Jaramogi Oginga Odinga Teaching and Referral Hospital (JOOTRH), 3 women are screened daily on average and yet the facility is a referral hospital for three provinces (Western, Nyanza and part of the Rift Valley). This number is low given the implications of late diagnosis and the advantages of early detection via screening. As such, the current study was designed to investigate the factors influencing uptake of cervical screening services at the JOOTRH.

## Methods

### Study design and study area

A cross-sectional survey was conducted between February and May 2012 at JOOTRH. This is a regional referral health facility located in Kolwa Location, Winam Division, Kisumu East District of Nyanza Province. Quantitative data was collected using structured questionnaires (see Additional file 1). The questionnaires aimed at determining the proportions of women who self-report having been screened for cervical cancer. The questionnaires further established in their opinion what could be the best way that could create more awareness about cervical screening. In addition, the respondents were asked whether they had a previous cervical screening and what prompted them to go for the screening. The other variables considered to be associated with the likelihood of reporting having a screen included socio-demographic factors, perceived susceptibility, severity and knowledge of sign and symptoms as described in the Health Belief Model (a psychological model that attempts to explain and predict health behaviors) [13].

### Sample size and sampling

Based on the available hospital records, a total of 25,991 women of child-bearing age (15–49 years) were seen at the Maternal and Child Health/Outpatient Department in 2010. Using a previously established formula [14], the total population was used to estimate the sample size (n = 384) for the study. A standard 10% for non-responses were added to this sample size to give a total of 424 respondents. The respondents (n = 424) were selected purposively since the group was dynamic. Every client who met the inclusion criteria (i.e. women above 18 years of age and less or equal to 49 years, seeking services at the JOOTRH during the study period, have at least one child or are pregnant) in each of the service delivery points was approached, consent sought and interviews performed until the required sample size of 424 was achieved.

### Data collection

Data were collected using interviewer-administered structured questionnaires. The questionnaires were translated to local dialects (*Dholuo* and Kiswahili) to enhance understanding during data collection and the responses were then back-translated to English.

### Ethical considerations

Ethical clearance was sought from the New Nyanza Provincial General Hospital Education and Research Committee in Kisumu, Kenya (Reference # NNPGH/ERC/9/12). Informed consent was sought from the study participants prior to data collection and confidentiality was maintained throughout the study.

### Data management and statistical analysis

Quantitative data collected through questionnaires was first verified to ensure completeness. Data forms were created with Epi Info version 7 (CDC, Washington DC, USA), verified by ensuring that the study identities were matching the data in the questionnaires and prior to exporting to Statistical Package for Social Scientists (SPSS) version 16.0 (IBM, USA), where both descriptive and inferential analyses were performed. Statistical significance of differences in proportions were determined using Chi-square analyses. Differences in age were determined using a Mann–Whitney U test. In order to identify predictors of reporting having had a cervical screen, the following parameters were regressed against self-reported uptake of cervical services: age, level of education, marital status, job status, income level and perceived severity of disease, level of knowledge on cervical cancer, and perceived susceptibility. *P*-values ≤0.05 were considered statistically significant.

## Results

### General and demographic characteristics of the study participants

A total of 424 respondents were interviewed from various reproductive health services delivery points (Table 1). Table 2 presents the demographic characteristics of the respondents. Out of the 424 respondents interviewed, 74 (17.5%) were screened while 350 (82.5%) were not screened. Median age between the groups was significantly different (*P* < 0.0001), given that those who were screened were generally older than those not screened. An additional chi-square test demonstrated that source of income (*P* < 0.0001), monthly income (*P* = 0.005) and level of education (*P* < 0.0001) significantly differed between the screened and not screened groups. However, the proportions of those in the different categories of the marital status were comparable between the screened and not screened groups (*P* = 0.132; Table 2).

**Table 1:** **Service delivery points of 424 respondents**

	Frequency	Percent
Antenatal Clinic	54	12.7
Antenatal Ward	18	4.2
Child Welfare Clinic	41	9.6
Family Planning Clinic	184	43.3
Female Medical Ward	35	8.2
Gynaecology Ward	18	4.2
Postnatal Ward	74	17.4
**Total**	**424**	**100.0**

**Table 2:** **Demographic characteristics of the study respondents (n = 424)**

Characteristic		Screened	Not screened	***P***
No. of participants		74	350	N/A
Age (Years)		29.0 (11.0)	26.0 (10.0)	**<0.0001** ^**a**^
Marital status	Married	55 (74.3)	246 (70.3)	0.132^b^
	Single	18 (24.3)	71 (20.3)	
	Divorced/Separated	0 (0.0)	4 (1.1)	
	Widowed	1 (1.4)	29 (8.3)	
Occupation/Source of income	White-collared	33 (44.6)	72 (20.5)	**<0.0001** ^**b**^
	Farming	4 (5.4)	22 (6.3)	
	Unskilled work	0 (0.0)	18 (5.1)	
	Daily wages	10 (13.5)	21 (6.0)	
	Business	10 (13.5)	37 (10.6)	
	Others	17 (23.0)	180 (51.5)	
Monthly income	<5,000	44 (59.5)	272 (77.7)	**0.005** ^**b**^
	5001-9,999	12 (16.2)	41 (11.7)	
	10,000–14,999	16 (21.6)	31 (8.8)	
	Don’t know	2 (2.7)	6 (1.8)	
Level of education	No formal education	0 (0.0)	29 (8.3)	**<0.0001** ^**b**^
	Primary	18 (24.3)	176 (50.3)	
	Secondary	35 (47.3)	96 (27.4)	
	Colleges	21 (28.4)	49 (14.0)	

Data are numbers (proportions). ^a^Statistical significance determined by Mann–Whitney U test. Age is in median years (interquartile range). ^b^Statistical significance determined by Chi-square analysis. Values in bold are statistically significant at *P* ≤ 0.05.

### Level of knowledge on cervical cancer and screening

Prior to determining the associations between level of knowledge and likelihood of being screened, a chi-square analyses between different knowledge levels against those screened and not screened was performed (Table 3). Results revealed that significant proportions of respondents were screened as their ability to respond correctly to questions about signs and symptoms of cervical cancer increased (*P* < 0.001; Table 3). A higher proportion (6/9; 66.7%) of those who gave 5 correct answers on the signs and symptoms for cervical cancer, were screened in the population. These proportions were higher relative to those that were knowledgeable about 3–4 correct answers (19/74; 25.7%), 1–2 correct answers (23/74; 31.1%) and no correct answers (26/74; 35.1%) (Table 3).

**Table 3:** **Distribution of study respondents (n = 424) based on their level of knowledge**

Characteristic		Screened	Not screened	***P***	OR	95% CI	***P***
No. of participants		74	350	N/A			
Level of knowledge on signs and symptoms	No correct response	26 (35.1)	242 (69.1)	<0.0001^b^	18.61	4.39-78.86	**<0.0001**
	1-2 correct responses	23 (31.1)	65 (18.6)		5.65	1.30-24.46	**0.021**
	3-4 correct responses	19 (25.7)	40 (11.4)		4.21	1.00-18.67	**0.050**
	5 correct responses	6 (8.1)	3 (0.9)		1.00	N/A	N/A

^b^Statistical significance determined by Chi-square analysis. Values in bold are statistically significant at *P* ≤ 0.05. Odds Ratios (OR) and 95% confidence intervals. The reference group in the logistic regression analyses was those who gave 5 correct signs and symptoms for cervical cancer. N/A = Not applicable.

Further logistic regression analyses demonstrated that respondents who had no knowledge about the signs and symptoms were about 18 times more likely to report not having been screened (OR, 18.61, 95% CI, 4.39-78.86; *P* < 0.0001; Table 3). The likelihood of not being screened decreased as the knowledge on the signs and symptoms increased (Table 3). Collectively, these data demonstrate that knowledge level on the signs and symptoms was an important determinant of reporting having had a cervical screen.

### Perceptions on severity and susceptibility to cervical cancer

In order to determine perception and susceptibility to cervical cancer, respondents were asked about their opinions on the seriousness of cervical cancer and how much they think it is a problem in their region. This was then compared with their tendency to present for cervical screening. There was a high likelihood of those who perceived cervical cancer as a serious disease to report having had a cervical screen. These women were significantly more likely to report having had a cervical screen (*P* < 0.0001; Table 4). Further logistic regression analysis demonstrated that those who said they didn’t know about the disease (OR, 26.84, 95% CI, 6.07-118.61, *P* < 0.0001) or were not aware about susceptibility to it (OR, 2.37, 95% CI, 1.10-5.08, *P =* 0.02) had a higher likelihood of not being screened for cervical cancer (Table 4).

**Table 4:** **Distribution of study respondents (n = 424) based on their perceived severity and susceptibility**

Perception		Screened	Not screened	***P-value***	OR	95% CI	***P-value***
No. of participants		74	350	N/A			
Seriousness of disease	Dont know	2 (2.7)	142 (40.6)	**<0.0001** ^**b**^	26.84	6.07-118.61	**<0.0001**
	Not serious	2 (2.7)	6 (1.7)		0.70	0.12-4.15	0.700
	Somewhat serious	2 (2.7)	23 (6.6)		5.83	1.27-26.65	**0.023**
	Very serious	68 (91.9)	169 (51.1)		1.00	N/A	N/A
Seriousness of disease in your area	Dont know	24 (32.4)	222 (63.4)	**<0.0001** ^**b**^	1.11	0.58-2.11	0.750
	Not serious	0 (0.0)	7 (2.0)		4.99	0.12-5.15	0.450
	Somewhat serious	15 (20.3)	31 (8.9)		0.58	0.27-1.25	0.164
	Very serious	35 (47.3)	90 (25.7)		1.00	N/A	N/A
Perceived susceptibility	Yes	11 (14.9)	24 (6.9)	**0.023** ^**b**^	1.00	N/A	N/A
	No	63 (85.1)	326 (93.1)		2.37	1.10-5.08	**0.020**

Data are n (%). ^b^Statistical significance determined by Chi-square analysis. Values in bold are statistically significant at *P* ≤ 0.05. N/A = Not applicable. Odds Ratios (OR) and 95% confidence intervals. The reference group in the logistic regression analyses was those who gave 5 correct signs and symptoms for cervical cancer.

### Cues to action

Results revealed that those who attended the child welfare clinic had a higher proportion represented in those that got screened for cervical cancer (*P* = 0.011). Those who attended the child welfare clinic were significantly more likely to report having a cervical screen (OR, 2.31, 95% CI, 1.17-3.93, *P =* 0.03) (Table 5).

**Table 5:** **Distribution of study respondents (n = 424) according to the service delivery points**

Characteristic		Screened	Not screened	***P-value***	OR	95% CI	***P-value***
No. of participants		74	350				
Antenatal Ward	Yes	4 (5.4)	14 (3.3)	**0.011** ^**b**^	0.67	0.19-2.41	0.54
Child Welfare Clinic	Yes	14 (19.0)	27 (6.4)		2.31	1.17-3.93	**0.03**
Family Planning Clinic	Yes	30 (40.6)	154 (36.5)		0.99	0.47-2.06	0.98
Female Medical Ward	Yes	8 (10.9)	27 (7.7)		0.65	0.24-1.78	0.40
Gynaecological Ward	Yes	4 (5.4)	14 (4.0)		0.67	0.19-2.41	0.54
Post-natal Ward	Yes	12 (16.2)	62 (17.7)		1.00	N/A	N/A

Data are n (%). ^b^Statistical significance determined by Chi-square analysis. Values in bold are statistically significant at *P* ≤ 0.05. N/A = Not applicable. Odds Ratios (OR) and 95% confidence intervals. N/A = Not applicable.

Additional analyses in which all the established predictive domains of knowledge of cervical cancer, perceived threat from cervical cancer, and service delivery point of origin of the respondent demonstrated that individuals with all combined predictive domains had an 11-fold chance of reporting having had a cervical screen (OR, 11.37, 95% CI, 7.21-18.393, *P =* 0.001). These demonstrate that combined approach can lead to an increased likelihood of having a cervical screen.

## Discussion

Cervical screening and early treatment of cervical cancer if detected, remains the most effective way to reduce the mortality associated with the disease. The study was conducted to establish the level of uptake of this very vital service at the JOOTRH by the targeted population in Kenya. The study also intended to explore factors associated with uptake of cervical screening services at this facility. Results demonstrated that out of a total of 424 respondents only 74 (17.5%) reported that they went for cervical screening. Knowledge level on the signs and symptoms was an important determinant for being screened for cervical cancer. In addition, those who said they didn’t know about the disease or who were not aware about susceptibility to it had a higher likelihood of not being screened for cervical cancer. Amongst the cues to action, being at the child welfare clinic was significantly associated with self-reported cervical screening.

Findings from the demographic characteristics in this facility reveal that the uptake is still significantly low at 17.5% as compared to the national target of 75% by the year 2009 [4]. A similar study carried out in South Africa, showed a figure of 18% [15]. In a similar study performed at Moi Teaching and Referral Hospital, Eldoret, Kenya, only 12.5% of the participants reported being screened [16]. Differences in the current study (17.5%) versus previous one (12.5%) could be attributed to the fact that the previous study was carried out at a time that the National Cervical Cancer Program had just been launched. As such, relatively less people could have been aware about it at the time. Furthermore, the previous study was embedded in a national program as opposed to the current hospital-based study. Such differences in study design could potentially lead to the observed differences between the current and the previous Kenyan study. A Knowledge, Attitude and Practices (KAP) survey done in Kenyatta National Hospital revealed a past Pap smear screening rate of 22% [8], which demonstrated a slightly higher proportion than that shown in the current study, possibly as a result of the presence of a more urban population and a higher socio-economic status. Another study performed in Voluntary Testing and Counseling (VCT) centers in Nairobi, Kenya [9] described an uptake rate of 14%. This is relatively lower than that of the current study and could be attributed to the fact that the previous uptake was based on a particular test (Pap smear) while in the current study, we included other cheaper methods of visual inspection using acetic acid (VIA) and visual inspection using Lugol’s iodine (VILLI). In addition, the current study relied upon self-reported cervical screening and not on actual values, a factor that may also be attributed to the variation in figures observed between the current and previous studies. Results further showed that age differed significantly between screened versus not screened while the marital status was an important predictor of uptake which is congruent with a study done in Britain [9] and a desk review done by Gannon and Dowling [11]. Other socio-economic factors, for example, having any form of employment, monthly income and level of formal education were significant determinants of uptake of this important service. These findings are consistent with those in studies carried out in India [17, 18] and at Kenyatta National Hospital (KNH), Kenya [8]. Increased socio-economic status place the women population in a better position economically and knowledge-wise thus increasing the likelihood of them seeking for cervical screening.

The current study further showed that out of the 424 respondents 286 (67.5%) have ever heard of cervical cancer, which is considerably higher than the figure described by the KNH study (51%) [8]. This discordance in results may be attributed to the differences in timing between the two studies in view of the improved communication, especially media coverage in Kenya as well as education rates. The level of knowledge on cervical cancer regarding its signs and symptoms, at risk group and prevention is low among the population under study. The 63.2% of the respondents had no knowledge (level 0) on the three aspects of the condition and only 2.1% had adequate knowledge (level 3) on the aspects of the condition. This was not much different from a South African study in which the figures were 65% (level 0) and 6% (level 3) [15]. This is of concern since analysis in the current study indicated that knowledge level on the signs and symptoms was an important determinant for being screened for cervical cancer (*P* < 0.0001). As knowledge on the signs and symptoms for cervical cancer increased, the respondents had a higher likelihood of being screened for cervical cancer, a phenomenon observed in previous studies in India [7, 12].

We also demonstrated that there was a high tendency of those who perceived cervical cancer as a serious disease to go for cervical screening. Most of those that perceived it to be serious were significantly represented in the groups who went for self-reported cervical screening. Further logistic regression analyses demonstrated that those who indicated that they didn’t know about the disease or were not aware about susceptibility to it had a higher likelihood of not being screened for cervical cancer. These observations were similar to a study carried out at the Moi Teaching and Referral Hospital [19] and support the Health Belief Model that the importance of perceived severity and susceptibility guides the decision to seek a service such as cervical screening. Increased perceived risk would thus enhance increased need to seek for cervical screening in this population.

Further findings in the current study showed a significant association between self-reported screening and being at the child welfare clinic which additionally increased the chances of one reporting having been screened. This is not in line with the findings of other studies which indicated that previous exposure to reproductive health services with possible health education of various topics increased awareness of cervical screening in an Indian population [17]. These differences could be attributed to a possibility that the health education given at the service delivery points at JOOTRH does not include cervical cancer. In our opinion, this is the first study that tried to demonstrate on the cues as part of the determinants of uptake of cervical cancer reported in an African set-up. As such, more studies need to be carried out in Africa to further assist in identifying additional cues for action for essential services such as cervical screening.

### Limitations

The current study had limitations in that it used a special population, that is, those seeking health services at the JOOTRH and thus were more likely to seek screening services. It also evaluated self-reports of screening which may not be entirely authentic.

## Conclusions

We have demonstrated that knowledge level on the signs and symptoms is an important determinant for being screened for cervical cancer. Those who indicated that they didn’t know about the disease or were not aware about susceptibility to it had a higher likelihood of not being screened for cervical cancer. Being at the child welfare clinic increased ones chances of having self-reported cervical screening. More emphasis should be on creating additional awareness about cervical screening at all service delivery points within the health facilities. This will lead to an enhanced knowledge and reduced morbidity and mortality associated with cervical cancer.

## Electronic supplementary material

Additional file 1: **Tools used to establish determinants of Cervical screening services uptake among 18–49 year old women seeking services at the Jaramogi Oginga Odinga Teaching and Referral Hospital, Kisumu, Kenya.** (DOC 86 KB)

## References

[1] Varughese J, Richman S (2010). Cancer care inequity for women in resource-poor countries. Rev Obstet Gynecol.

[2] Canavan TP, Doshi NR (2000). Cervical cancer. Am Fam Physician.

[3] Safaeian M, Solomon D, Castle PE (2007). Cervical cancer prevention–cervical cancer screening: science in evolution. Obstet Gynecol Clin North Am.

[4] MOH (2005). Ministry of Health: National Cervical Cancer Prevention Programme.

[5] MOH (2010). Ministry of Health: Review of the 2004–2008 reproductive health research agenda proposed 2010–2014 research agenda.

[6] Christou A, Katzenellenbogen JM, Thompson SC (2010). Australia’s national bowel cancer screening program: does it work for indigenous Australians?. BMC Public Health.

[7] WHO/ICO (2010). Human Papilloma Virus and related cancers in Kenya. ICO Information Centre on HPV and Cancer.

[8] Gichangi P, Estambale B, Bwayo J, Rogo K, Ojwang S, Opiyo A, Temmerman M (2003). Knowledge and practice about cervical cancer and Pap smear testing among patients at Kenyatta National Hospital, Nairobi. Kenya Int J Gynecol Cancer.

[9] Rositch AF, Gatuguta A, Choi RY, Guthrie BL, Mackelprang RD, Bosire R, Manyara L, Kiarie JN, Smith JS, Farquhar C (2012). Knowledge and acceptability of pap smears, self-sampling and HPV vaccination among adult women in Kenya. PLoS One.

[10] Sutton S, Rutherford C (2005). Sociodemographic and attitudinal correlates of cervical cancer screening uptake in a national sample of women in Britain. Soc Sci Med.

[11] Gannon M, Dowling M (2008). Increasing the uptake of cervical cancer screening programmes. Br J Nurs.

[12] Bernatsky S, Hudson M, Pope J, Markland J, Robinson D, Jones N, Docherty P, Abu-Hakima M, Leclerc S, Dunne J, Smith CD, Sutton E, Khalidi N, Mathieu JP, Masetto A, Ligier S, Kaminska E, Baron M, Canadian Scleroderma Research Group (2009). Reports of abnormal cervical screening tests in systemic sclerosis. Rheumatology (Oxford).

[13] Basavanthappa BT (2008). Health Belief Model. Community Health Nursing.

[14] Hsieh FY, Bloch DA, Larsen MD (1998). A simple method of sample size calculation for linear and logistic regression. Stat Med.

[15] Hoque M, Hoque E, Kader SB (2008). Evaluation of cervical screening program at a rural community of South Africa. East Afr J Public Health.

[16] Were E, Nyaberi Z, Buziba N (2011). Perceptions of risk and barriers to cervical cancer screening at Moi Teaching and Referral Hospital (MTRH), Eldoret, Kenya. Afr Health Sci.

[17] Dhamija S, Sehgal A, Luthra UK, Sehgal K (1993). Factors associated with awareness and knowledge of cervical cancer in a community: implication for health education programmes in developing countries. J R Soc Health.

[18] Nene B, Jayant K, Arrossi S, Shastri S, Budukh A, Hingmire S, Muwonge R, Malvi S, Dinshaw K, Sankaranarayanan R (2007). Determinants of womens participation in cervical cancer screening trial, Maharashtra, India. Bull World Health Organ.

[19] Were E, Nyaberi Z, Buziba N (2010). Integrating cervical cancer and genital tract infection screening into mother, child health and family planning clinics in Eldoret, Kenya. Afr Health Sci.

[CR20] The pre-publication history for this paper can be accessed here:http://www.biomedcentral.com/1472-6963/14/335/prepub

