# Supplementary material for: Determinants of Cervical screening services uptake among 18–49 year old women seeking services at the Jaramogi Oginga Odinga Teaching and Referral Hospital, Kisumu, Kenya
Source: BMC Health Serv Res. 2014 Aug 6;14:335. doi: 10.1186/1472-6963-14-335 (PMC4271847; doi:10.1186/1472-6963-14-335)
Supplement: Supplementary file 1 — Additional file 1: Tools used to establish determinants of Cervical screening services uptake among 18–49 year old women seeking services at the Jaramogi Oginga Odinga Teaching and Referral Hospital, Kisumu, Kenya. (DOC 86 KB) [file 12913_2013_3442_MOESM1_ESM.doc]

# A: INFORMED CONSENT FORM (ENGLISH)

**Information sheet**

The following information is to enable you to give voluntary, informed consent to participate in this study. Please read the information carefully before signing the consent form (part B).To be verbally read for those who are not able to read.

**Study title**: Determinants of cervical cancer screening services uptake among 18-49 year old women seeking services at the NNPGH.

**Investigators Names**: Morema Everlyne Nyanchera

**Address**

Nyanza Provincial General Hospital,

P.O. Box 849,

Kisumu.

Tel: +254-721 262 748

**Aim and Significance of the study**

This study aims at describing and analyzing the factors of health care seeking behaviour among women of child bearing age who are at risk of acquiring cervical cancer with regards to the screening services offered at the NNPGH. The study will also seek to establish the knowledge, attitudes and practices among the target group. This therefore will be a tool for cervical cancer prevention policy makers and program planners to enhance uptake of this essential service.

**What participation will involve**

Upon enrolment in the study, you will be asked detailed questions on demographic data and socioeconomic activities. This information will be recorded onto forms. In addition a field assistant will ask you questions concerning the cervical cancer and screening services. The interview will take at most 10 minutes. As a participant in this study, you will be required to give honest information to their level best. Participation is voluntary and refusal to participate will involve no penalty. You may withdraw from participating in this study at any time without giving reasons. There are no foreseeable risks and immediate benefits for participating in this study. The findings from this study will help improve service provision.

**Data Security**

All information you provide us will remain confidential. Only the study team will have this information and will be treated with confidentiality unless your express permission is obtained. This will not affect services you are receiving.

CONSENT FORM

Please read the previous information sheet (or have the information read to you) carefully before completing and signing this consent form. Should you have any questions about the study please feel free to ask the investigator prior to signing your consent

**Consent Form for the Study**

Determinants of cervical cancer screening services uptake among 18-49 year old women seeking services at the NNPGH.

Investigators name: Morema Everlyne Nyanchera

Nyanza Provincial General Hospital,

P.O. Box 849,

Kisumu.

Tel: +254-721 262 748

**FOR COMPLETION BY PARTICIPANTS**

I have read the following sheet concerning this study and I understand what will be required of me if I take part in the study.

Any questions regarding this study have been answered by

………………………………………………………………………………………………

I understand that at any time I may withdraw from the study without giving a reason and this will not affect the care am receiving at the hospital.

**I AGREE TO TAKE PART IN THE STUDY:**

Name of participant:………………………………………………………………………

Signed………………………………………………….. (Or thumb print)

Date:……………………………………………………..

# B: QUESTIONNAIRE FOR INTERVIEW (ENGLISH VERSION)

**IDENTIFICATION INFORMATION**

Unique number

Interviewer:

Service delivery point

1. Post natal
2. Fp clinic
3. Cervical cancer screening room
4. Immunization room
5. Gynaecology ward

Ethnicity:

Religion:

1. Hindu

2. Buddhist

3. Muslim

4. Christian

5. Other specify ……………………….

**SECTION 1-SOCIO DEMOGRAPHIC INFORMATION**

**Please circle the options below**

**Q.1. What is your age please?**

Age: __________ years

**Q. 2 What is the highest level of education that you have attained?**

DON'T READ THE OPTIONS BELOW. SINGLE CODE

| Illiterate | [1] |
| --- | --- |
| No formal Education but can read and write | [2] |
| Primary education | [3] |
| Secondary | [4] |
| Tertiary | [5] |
| Occupational Education | [6] |
| Any other (Specify:______________) | [ ] |

**Q. 3 Which type of area do you come from?**

Urban [1]

Rural [2]

Don’t know [98]

**QQ. 5 What is your current marital status?**

Unmarried………………. [1]

Married………………….. [2]

Divorced…..……………. [3]

Widowed……………….. [4]

Q. 6 Have you ever had a baby?

Yes

No (skip Q 7)

Q. 7. What is the total number of living children that you have?

Sons: __________

Daughters: _______

Don’t have any children………… [98]

Q.8 At what age did you get your first child?

Below 18 years (1)

Between 18- 24 years (2)

Above 24 years (3)

Q. 9. Have you ever used any hormonal contraceptive method (Depo Provera, POPs, COCs, implants, hormone containing IUCDs)?

Yes (1)

No (2)

Q. 10. For how long have you used the method?

Q.11 What is your job status?

| Employed | [1] |
| --- | --- |
| Unemployed | [2] |
| Housewife | [3] |
| Farmer | [4] |
| Professional | [5] |
| Unskilled worker | [6] |
| Daily wages  Business lady  Any other (please specify) | [7]  [ ] |

Q. 12. What is your personal monthly Income (KShs)?

| <5000 | 1 |
| --- | --- |
| 5000 – 9999 | 2 |
| 10000 to 14999 | 3 |
| More than 15000 | 4 |
| Don’t know | 98 |

Q.13 Please tell me the number of your family members? (Just enter the number in the box provided)

| Adults | ( ) |
| --- | --- |
| Children | ( ) |
| Total | ( ) |
|  |  |

**SECTION 2 – KNOWLEDGE AND PERCEPTION ON CERVICAL CANCER**

Q1. Had you ever heard of cervical cancer?

Yes [1]

No [2]

Q2. If yes, what was the source of information?

TV [1]

Radio [2]

### Billboards [3]

Newspapers/magazine [4]

Doctor [5]

Pharmacy [6]

Friends/Relatives [7]

Religious leaders [8]

Teachers [9]

Other (specify …..) [10]

Q3. In your opinion how serious is cervical cancer?

Very serious [1]

Somewhat serious [2]

Not very serious [3]

Don’t know [98]

Q 4. How serious a problem do you think cervical cancer is in your region?

Very serious [1]

Somewhat serious [2]

Not very serious [3]

Don’t know [98]

Q5. Please tell me some of the sign and symptoms of cervical cancer?

**DON'T READ THE OPTIONS BELOW- MULTIPLE RESPONSE EXPECTED**

Abdominal pain [1]

Vaginal bleeding [2]

Dyspareunia (Coital pain) [3]

Coughing blood [4]

Severe Headache [5]

Nausea [6]

Loss of weight [7]

Abnormal vaginal discharge [8]

Don’t know [98]

N/A

Q 6. How can a person get cervical cancer?

Using hormonal contraceptives [1]

Having multiple sexual partners [2]

Familial tendency [3]

Smoking [4]

Touching items in public places e.g. handles, knobs [5]

Other (please specify)… [6]

Don’t know [98]

Q 7. How can a person prevent getting cervical cancer? (Multiple answers can be expected)

Be faithful to ones partner [1]

Avoiding early debut to sexual activities [2]

Using a condom [3]

Early diagnosis and treatment through screening [4]

Having fewer children

Male partner circumcision

Other (please explain) [5]

Don’t know [98]

Q 8. In your opinion, who can be infected with cervical cancer? (Multiple answers expected)

Anybody [1]

Women of childbearing age

Only poor people [2]

Commercial sex workers [3]

Only alcoholics [4]

Old women [5]

Only people living with HIV/AIDS [6]

Only people who have been in prison [7]

Other (please explain) [8]

Don’t know

**SECTION 3 – HEALTH SEEKING BEHAVIOUR**

Q 1. How often do you generally seek health care at a clinic or hospital? (Check one.)

Twice a year [1]

Once a year [2]

Less than a year but twice in the past [3]

Once in the past 5 years [4]

Not in the past year [0]

Q 2. Where do you usually first seek care when sick?

Governemnt Hospital [1]

Private clinic [2]

Traditional healer [3]

Pharmacy [4]

Don’t know [98]

Q 4. Are you satisfied with services you are receiving currently?

Satisfied [1]

Not satisfied [2]

Don’t know [98]

Q5. What is the best way that could be used to make you more aware about cervical cancer?

------------------------------------------------------------------------------------------------------------

Q6. Have you ever been screened for cervical cancer?

Yes (1)

No (skip to question 10) (2)

Q7. What made you go for screening?

------------------------------------------------------------------------------------------------------------

Q8. a) Do you know of any cultural issues associated with cervical cancer or the screening methods for cervical cancer?

Yes (1)

No (2)

b)If YES which one?------------------------------------------------------------------------------------------

Q9. Do you know that screening services are offered in this hospital**?**

Yes [1]

No [2] Skip to question 14

Q10. What do you think about the service offered?

Excellent [1]

Good [2]

Poor [3]

Don’t know [98]

Q11. What do you think about the behaviour of health workers offering these services?

Excellent [1]

Good [2]

Poor [3]

Don’t know [98]

Q12. What do you think of the procedure of being screened for cervical cancer

----------------------------------------------------------------------------------------------

Q13. Is the cervical cancer screening at the NNPGH affordable?

Q14. In your experience what is the waiting time for this clinic before receiving required services?

0-15 minutes [1]

15-30 minutes [2]

>30 minutes [3]

>1 hour [4]

Don’t know [98]

Q.15. How long does it take to reach these hospital?

| <1 hour | [1 ] |
| --- | --- |
| >1 hour | [ 2] |
| Don’t know | [98] |

***Thank you for your time.***
